# Monitoring the immune response to vaccination with an inactivated vaccine associated to bovine neonatal pancytopenia by deep sequencing transcriptome analysis in cattle

**DOI:** 10.1186/1297-9716-44-93

**Published:** 2013-10-07

**Authors:** Wiebke Demasius, Rosemarie Weikard, Frieder Hadlich, Kerstin Elisabeth Müller, Christa Kühn

**Affiliations:** 1 Institute for Genome Biology, Leibniz Institute for Farm Animal Biology (FBN), Wilhelm-Stahl-Allee 2, 18196 Dummerstorf, Germany; 2 Clinic for Ruminants and Swine, Department of Veterinary Medicine, Freie Universität Berlin, Königsweg 65, 14163 Berlin, Germany

## Abstract

Bovine neonatal pancytopenia (BNP) is a new fatal, alloimmune/alloantibody mediated disease of new-born calves induced by ingestion of colostrum from cows, which had been vaccinated with a specific vaccine against the Bovine Virus Diarrhoea Virus (BVDV). The hypothesis of pathogenic MHC class I molecules in the vaccine had been put up, but no formal proof of specific causal MHC class I alleles has been provided yet. However, the unique features of the vaccine obviously result in extremely high specific antibody titres in the vaccinated animals, but apparently also in further molecules inducing BNP. Thus, a comprehensive picture of the immune response to the vaccine is essential. Applying the novel approach of next generation RNA sequencing (RNAseq), our study provides a new holistic, comprehensive analysis of the blood transcriptome regulation after vaccination with the specific BVDV vaccine. Our RNAseq approach identified a novel cytokine-like gene in the bovine genome that is highly upregulated after vaccination. This gene has never been described before in any other species and might be specific to ruminant immune response. Furthermore, our data revealed a very coordinated immune response to double-stranded (ds) RNA or a dsRNA analogue after vaccination with the inactivated single-stranded (ss) RNA vaccine. This would suggest either a substantial contamination of the vaccine with dsRNA from host cells after virus culture or a dsRNA analogue applied to the vaccine. The first option would highlight the potential risks associated with virus culture on homologous cells during vaccine production; the latter option would emphasise the potential risks associated with immune stimulating adjuvants used in vaccine production.

## Introduction

Vaccination regimes are a powerful strategy to protect our animal populations against microbial diseases (e.g., [1]). However, application of vaccination regimes requires a comprehensive knowledge about all potential vaccination effects. Bovine neonatal pancytopenia (BNP) is a new disease of new-born calves characterised by extreme haemorrhages, thrombocytopenia, leukocytopenia, and cellular depletion of the bone marrow [2]. BNP ends lethally in the vast majority of cases, and no specific treatment is available. Recent studies convincingly revealed that BNP is an alloimmune/alloantibody mediated disease induced by ingestion of colostrum from cows vaccinated with a specific vaccine (PregSure®) against the Bovine Virus Diarrhoea Virus (BVDV) [3-5]. This inactivated vaccine directed against a single stranded (ss)RNA virus is distinguished by an extremely high production of specific BVDV antibodies, which is potentially due to the unique adjuvant included in the vaccination dose. Alloantibodies induced after vaccination with the PregSure® BVDV vaccine bind to MHC class I cell surface proteins of leukocytes and also of the Madin-Darby bovine kidney (MDBK) cell line, which had been used for production of the specific BVDV vaccine [6,7]. Thus, the hypothesis is that contaminating MHC class I antigens from the bovine MDBK cell line in the vaccine act as alloantigens and elicit the production of the pathogenic alloantibodies. However, the exact pathogenesis of BNP is still not fully elucidated. For example, experimental vaccinations in several studies showed a much higher proportion of individuals with alloantibodies than reported BNP cases in the population relative to the large number of vaccinated individuals [8]. Furthermore, no formal proof of a specific causal MHC class I allele has been provided yet. The specific nature of the vaccine composition can be elucidated by comprehensive knowledge about quantitative and structural regulation of the blood transcriptome after vaccination with the specific BNP-associated vaccine, which will provide novel insights into the immune response to the vaccine.

Deep sequencing of a transcriptome by next generation RNAseq offers the tool for a precise and truly holistic analysis of the expressed loci within cells and tissues [9]. Recent studies showed that it outperforms previous methods for transcriptome analysis due to its large dynamic range and low technical variance [10]. In addition, RNAseq is not restricted to the known genome annotation but enables identification of novel, previously unknown functionally relevant loci in the genome as recently exemplified by the discovery of a novel human interferon gene [11]. This property to add information to an existing genome annotation is valuable especially in genomes with no high-quality annotation like in many livestock species including cattle.

Recently, a transcriptome analysis of peripheral blood mononuclear cells in calves using deep sequencing reported a major IFNγ/IL22 response to vaccination directed against Mycobacterium bovis [12]. Furthermore, KEGG pathways *Cytokine – cytokine receptor interaction*, *Cell cycle*, *Prion diseases* and *p53 signalling pathway* were significantly modulated. This demonstrates that RNAseq experiments are a useful tool for monitoring the immune response to vaccination.

To our knowledge, we present the first whole blood transcriptome analysis of a livestock species in response to a virus-based vaccine applying deep RNA sequencing. The results provide a comprehensive catalogue of the immune response to the specific vaccine indicating a major reaction to RNA virus infection and an activation of T cell response. In addition, a new cytokine-like gene with strong protein-coding potential was identified for the first time, which was highly upregulated after vaccination, and which has not yet been described in cattle or any other species before.

## Materials and methods

### Animals

All experimental procedures were carried out according to the German animal care guidelines and were approved and supervised by the relevant authorities of the State Mecklenburg-Vorpommern, Germany (State Office for Agriculture, Food Safety and Fishery Mecklenburg-Western Pommerania (LALLF M-V), 7221.3-2.1-005/11). The study included 12 lactating and non-lactating cows aged three to five years. Except one Holstein cow, all individuals were F_2_ cows from a German Holstein × Charolais crossbred population [13]. All cows were kept under the same dairy cow conditions on the experimental farm of the FBN Dummerstorf. All cows had received a basic double vaccination with an inactivated BVDV vaccine (PregSure®, Pfizer, Berlin, Germany) according to the manufacturer’s recommendations and at least one booster vaccination 15 months prior to our experiment. Thus, the immune response monitored in our experiment is a recall response to a previously encountered vaccine. Four of the cows had calves that had developed a clinical BNP. For this study, jugular blood was taken immediately prior a further booster vaccination with PregSure® and 14 days later. The time point day 14 was selected due to the documented relevance of alloantibodies for BNP and because antibody production after a PregSure® booster vaccination could be assumed to have reached a plateau at this date [14]. After sampling, 2.5 mL blood was immediately transferred to PAXgene blood RNA tubes (PreAnalytiX, Hombrechtikon, Switzerland). Samples were frozen and stored at −80 °C according to the manufacturer’s instructions until further processing.

For RT-PCR confirmation of RNAseq data regarding XLOC_032517, tissue samples and blood samples from four additional F_2_-cows from the German Holstein × Charolais crossbred population were analysed. All individuals had not received a vaccination with PregSure®, but had been vaccinated with an alternative inactivated BVDV vaccine. Tissue samples were taken immediately after slaughter and snap frozen in liquid nitrogen and stored at −80 °C until further processing.

### Sample preparation

Whole blood RNA was isolated by the PAXgene Blood RNA Kit (PreAnalytiX, Hombrechtikon, Switzerland) according to the manufacturer’s instructions and stored at −80 °C. Residual genomic DNA was carefully eliminated by on-column digestion using twice the amount of RNAse-free DNase I solution the manufacturers recommended in the protocols. The samples were monitored for RNA concentration with the NanoDrop 1000 system (Peqlab, Erlangen, Germany). RNA integrity was determined on the Bioanalyzer 2100 (Agilent, Böblingen, Germany). Potential sample contamination with genomic DNA was meticulously checked by PCR with genomic primers according to [15]. Those samples showing traces of contamination were again treated with DNAse I and purified according to the RNAeasy Min Elute Cleanup protocol (Qiagen, Hilden, Germany) to carefully remove all residual DNA. Only RNA samples without detectable DNA contamination were used for further processing in RNAseq and locus-specific RT-PCR experiments.

Total RNA from tissue samples was extracted as has been described previously [16]. Madin-Darby bovine kidney cells (MDBK) were grown in Eagle’s Minimal Essential Medium (EMEM) (Sigma-Aldrich Chemie, Steinheim, Germany) supplemented with 2 mM L-glutamine (Biochrom AG, Berlin, Germany), 1% non-essential amino acids (NEAA) (Biochrom AG, Berlin, Germany) and 10% heat-inactivated fetal calf serum (FCS) (PAN-Biotech GmbH, Aidenbach, Germany). Cells were maintained at 37 °C and 5% CO2. Total RNA was prepared from the MDBK-cells using Trizol (Invitrogen, Darmstadt, Germany). The RNA pellet was resuspended in 50 μL RNase-free water and stored at −80 °C until further processing. Check for DNA contamination and DNase treatment of total RNA from tissues and MDBK cells were performed as described for blood samples.

### Library preparation and sequencing

RNAseq libraries were prepared from 1 μg total RNA using the Illumina TruSeq RNA library preparation kit (Illumina, San Diego, USA) with indexed adapter sequences to enable sample multiplexing during cluster generation and sequencing by synthesis. For each individual, two libraries were prepared: from sampling before and 14 days after vaccination resulting in a total of 24 different TruSeq RNA libraries for sequencing. The RNAseq libraries were monitored for insert size with the Bioanalyzer 2100 (Agilent, Böblingen, Germany) and for highly repetitive sequences by cloning a subset of the libraries into a plasmid vector and sequencing of 40 randomly selected clones from each library.

Taking advantage of the index adaptors, individual mixes for each lane of the flow cells were prepared for sequencing by pooling libraries from 12 samples for each mix. Mixes were equally distributed on three flow cells to avoid technical bias of results. Paired-end sequencing with 2 × 61 cycles was performed on an Genome Analyzer GAIIx (Illumina, San Diego, USA) using a PhiX control. The resulting reads were demultiplexed using CASAVA v1.8. All demultiplexed reads of one sample from the different mixes and flow cells were merged into a single fastq file and checked for quality (base quality scores, adaptor contamination, repetitive sequences) using FastQC [17]. The fastq files passing quality threshold served as input for further analyses.

### Sequence assembly and locus annotation

Reads were aligned to the bovine reference genome UMD3.1 [18] using Bowtie/TopHat 2.03 [19] options. Tophat enables spliced read alignments. For guided alignment options, we supplied TopHat with the bovine gene model annotation from Igenome ([20], NCBI version, accession date 12/06/2012). The guided alignment option employs the reference annotation in a first alignment step using Bowtie to map reads against a virtual transcriptome generated from the annotation data and subsequently converts the mapped reads to genome mapping. The remaining reads failing to map to the virtual genome will then be further processed for spliced alignment against the genome. This strategy takes advantage of the existing annotation, but keeps also aligned reads mapping to previously unannotated transcription units of the genome.

The resulting BAM file from read alignment was filtered using SAMtools [21] for reads that showed more than two mismatches to the reference genome. Furthermore, for those reads with more than one reported alignment, only the alignment with the lowest query hit index was kept to avoid multiple counting of reads during expression analysis.

The filtered BAM file was submitted to transcript assembly using Cufflinks 2.02 options [19]. Each sample was first analysed individually for transcript assembly. The Igenome gene annotation (see above) was provided to guide the assembly. This enables the output of novel genes and isoforms in addition to the provided reference transcripts. The resulting .gtf-files containing the information on transcript assembly for each sample were merged using Cuffmerge with the Igenome reference annotation and the bovine Ensembl gene annotation release 66 [22]. This final .gtf-file was used for locus and transcript quantification using Cuffdiff 2.02 with the bovine UMD3.1 as reference genome assembly. Transcript and locus assemblies were visualised for inspection of the BAM files and the final annotation files with the Integrative Genomics Viewer [23].

### Differential expression analysis

The estimated number of fragments originating from each locus in the final annotation file was obtained using the Cuffdiff option of Cufflinks. The resulting read-group-tracking file was filtered for the estimated fragment count per locus for each individual and each time point. This served as input for tests of differential expression using edgeR [24]. In contrast to Cuffdiff, edgeR provides the option to include systematic effects, e.g., vaccination or animal effect in the model for differential expression by fitting linear models. To test for differential locus expression, a negative binomial distribution of read counts was assumed. Only loci with an expression level exceeding 0.1 counts per million reads (cpm) in each of the 24 samples were included. After calculation of the normalisation factor, the effect of the vaccination on differential gene expression was calculated with the model: counts = individual + treatment. Tests for statistical significance included accounting for multiple testing by calculating the false discovery rate (FDR) according to [25]. Only differences in expression with a significance threshold of q < 0.05 were considered statistically significant.

### Pathway analysis

For pathway analysis, only loci were included with a gene annotation and a statistically significant differential expression (q < 0.05) prior vs. after vaccination. The initial gene annotation from the transcript assembly process in Cufflinks was supplemented by BLAST search of sequences from unknown loci with the bovine NCBI Refseq sequences (accession date 21/11/2012). The final list of differentially expressed genes was analysed for affected pathways using GOseq [26] and Ingenuity pathway analysis [27]. For GOseq analysis, gene acronyms were translated into Ensembl IDs using the Biomart tools [28]. Due to the poor functional annotation of the bovine genome, we used the human Ensembl annotation for pathway analysis in GOseq. In the GOseq analysis, the length bias characteristic for RNAseq data is accounted for by using the Wallenius distribution to approximate the true null distribution. GOseq was subsequently applied to test KEGG pathways [29] for over- or underrepresentation in the set of differentially expressed genes. The respective *p*-value for over- or underrepresentation is calculated from the null distribution. The significant KEGG pathway maps were inspected for significantly differentially expressed genes.

Ingenuity pathway analysis was applied to identify biological functions, canonical pathways, networks and upstream regulators involved in the response to vaccination with PregSure®. For this purpose, the list of differentially expressed genes was investigated including the respective log_fold change_ to indicate the direction and quantity of differential expression. For identification of activation or inhibition of upstream regulators, a threshold for the activation z-score calculated in the IPA analysis of |z-score| > 2 was applied.

### RT-PCR confirmation of RNAseq results

The structure and expression of the novel locus XLOC_032517 were confirmed by locus-specific RT-PCR analysis. RNA from blood samples were investigated from all animals prior and after vaccination. In addition to individuals vaccinated with PregSure®, expression of XLOC_032517 was monitored in blood samples from three animals of the Charolais × German Holstein resource population, which had not been vaccinated with the specific BVDV vaccine. Furthermore, also a collection of tissues from a cow of the F_2_ resource population not vaccinated with the specific BVDV vaccine and also the MKBK cell line were investigated for XLOC_032517 expression.

One primer pair (TC_85490_F1/ TC_85490_R2, Additional file 1) was designed to amplify all exons of the XLOC_032517 transcript under the following PCR conditions: initial denaturation at 94 °C, 35 cycles of amplification at 62 °C annealing and 45 s for extension. The obtained PCR fragments were excised from the agarose gel, purified using the Nucleospin Extract II kit (Macherey and Nagel, Düren, Germany) and sequenced on a capillary sequencer (ABI310, Life Technologies, Darmstadt, Germany; MEGABACE 1000, GE Healthcare Europe, Freiburg, Germany). The obtained sequences were aligned to the sequence of XLOC_032517 as obtained by RNAseq using Bioedit [30].

Differential quantitative expression of XLOC_032517 was confirmed by quantitative real time RT-PCR using primers TC_85490_F3/ TC_85490_R3 essentially as described by [15] except for cDNA synthesis performed with oligo dT and random hexamer primers. Two reference genes (EIF3K, MTG1; Additional file 1) for normalisation were obtained from the RNAseq data by selecting loci with low normalised variance of cpm values across all samples and with expression levels in range with XLOC_032517. Statistical analysis of quantitative expression between time points was carried out with the SAS MIXED procedure analogous to the analysis of the deep sequencing data including the time point relative to vaccination as fixed and the animal as random effect in the model. Statistical analysis of differential quantitative expression between the 12 animals vaccinated with PregSure® (sample from day 0 of our experiment) and three cows that had not encountered PregSure® vaccination was calculated using SAS GLM.

## Results

### Read mapping

After demultiplexing and merging of reads, 33.1 – 45.7 million paired-end fragments (two reads per fragment) were obtained per sample (Table 1) amounting to a total of 953 259 252 million fragments for the entire experiment. After alignment and filtering, 57.8 – 79.7 million reads per sample (a total of 1 665 651 857 reads for the entire experiment) were submitted to further analysis. This dataset represented 85.1-89.0% of all reads indicating a high proportion of mapped reads and a high uniformity between samples for both, the number of mapped reads and also the percentage of mapped reads relative to all reads.

**Table 1:** Overview of the alignments of reads per sample.

**Individual**	**Sampling**	**Breed**	**Total number of fragments**	**Uniquely counted, mapped reads with < 3 mismatches**	**% of reads uniquely mapping, < 3 mismatches**	**% of reads mapping to globin clusters**
cow 1	before vacc	Cha × GH F_2_	44 776 690	77 918 387	87.01	1.04
cow 1	after vacc	Cha × GH F_2_	44 627 365	78 360 907	87.79	2.01
cow 2	before vacc	Cha × GH F_2_	43 990 812	77 993 272	88.65	0.07
cow 2	after vacc	Cha × GH F_2_	44 636 963	77 742 662	87.08	0.23
cow 3	before vacc	Cha × GH F_2_	40 798 596	71 377 966	87.48	0.10
cow 3	after vacc	Cha × GH F_2_	37 816 325	65 542 541	86.66	0.11
cow 4	before vacc	Cha × GH F_2_	35 447 535	61 851 369	87.24	0.18
cow 4	after vacc	Cha × GH F_2_	39 030 040	69 141 268	88.57	0.16
cow 5	before vacc	GH	34 889 160	61 093 146	87.55	0.40
cow 5	after vacc	GH	33 543 345	59 711 466	89.01	1.33
cow 6	before vacc	Cha × GH F_2_	42 232 327	73 237 641	86.71	0.10
cow 6	after vacc	Cha × GH F_2_	41 569 088	71 882 069	86.46	0.26
cow 7	before vacc	Cha × GH F_2_	40 572 312	70 148 894	86.45	3.06
cow 7	after vacc	Cha × GH F_2_	44 155 006	77 074 387	87.28	0.92
cow 8	before vacc	Cha × GH F_2_	35 704 593	61 371 734	85.94	0.51
cow 8	after vacc	Cha × GH F_2_	37 375 624	65 532 890	87.67	0.86
cow 9	before vacc	Cha × GH F_2_	33 125 544	57 825 014	87.28	0.37
cow 9	after vacc	Cha × GH F_2_	38 731 113	67 755 124	87.47	0.05
cow 10	before vacc	Cha × GH F_2_	36 740 555	62 541 431	85.11	1.10
cow 10	after vacc	Cha × GH F_2_	38 024 182	67 142 881	88.29	7.14
cow 11	before vacc	Cha × GH F_2_	36 549 423	63 893 567	87.41	2.17
cow 11	after vacc	Cha × GH F_2_	39 502 827	69 593 787	88.09	0.56
cow 12	before vacc	Cha × GH F_2_	45 701 139	79 680 037	87.18	0.16
cow 12	after vacc	Cha × GH F_2_	43 718 688	77 239 417	88.34	1.18
Total			953 259 252	1 665 651 857		

*Cha*, Charolais; *GH,* German Holstein; *vacc,* vaccination.

### Transcript annotation and quantification

Analysis of expression revealed that 28 690 loci exceeded an expression level threshold of 0.1 cpm (counts per million reads) in at least one sample, and 18 181 loci exceeded this threshold in all samples. 4596 (25.3%) of those loci with expression level > 0.1 cpm in all samples had no previous locus annotation in the Igenome NCBI annotation file.

The five loci with the highest cpm across all samples and time points were serine dehydratase (SDS), ribosomal S18 RNA (RN18S1), CD74 molecule, major histocompatibility complex (class II invariant chain, CD74), bovine major histocompatibility complex class I (BoLA) and β2 microglobulin (B2M), the latter three representing major determinants of the histocompatibility complex in cattle. However, it has to be considered that the cpm value does not take transcript length into account, which results in an up-bias of longer transcript compared to short transcripts.

Human transcriptome analysis by RNAseq is impaired by a high proportion of reads mapping to the human globin cluster [31]. Thus, a specific depletion of the RNA samples for globin sequences to enhance the complexity of the human RNAseq libraries is required. Initial Sanger sequencing of individual cloned fragments from our bovine blood RNAseq libraries identified only single clones containing sequences with homology to the bovine globin clusters (data not shown). After sequencing the RNAseq libraries on the Illumina Genome Analyzer GAIIx, we looked for reads mapping to the bovine α haemoglobin cluster on BTA25 (region 190 kb – 219 kb) or to the β haemoglobin cluster on BTA15 (region 48.990 Mb – 49.080 Mb). The percentage of reads mapping to these chromosomal areas [0.07 to 7% of all reads (Table 1)] is substantially lower compared to human studies (60%, [32]) and indicates that the complexity of the bovine RNAseq libraries is not compromised by a huge proportion of reads generated from the bovine globin clusters.

### Differential expression due to vaccination

**Figure 1:**
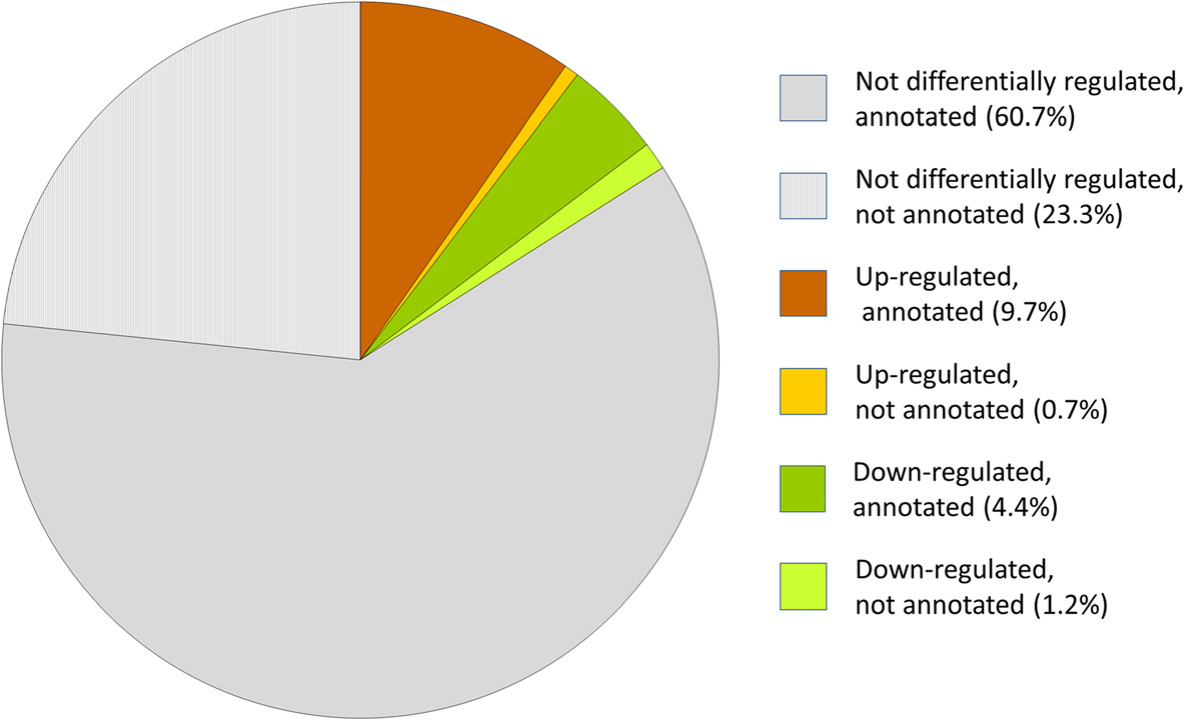
**Annotation and differential expression of transcribed loci within the bovine blood transcriptome.** All 18 181 transcripts identified in the bovine blood transcriptome with at least 0.1 cpm in all samples were investigated. Annotated and novel (= not annotated) transcripts are depicted regarding status of regulation after vaccination with a specific BVDV vaccine.

Analysis of the vaccination effect on blood RNA expression yielded 2901 differentially expressed loci at a significance threshold of q < 0.05 (Figure 1, Additional file 2). 2578 of the significantly differentially expressed loci had an official annotation or could be identified by BLAST search against the NCBI Refseq data set. For 323 differentially expressed loci at a q < 0.05 threshold (11.1% of all differentially expressed loci), there was no bovine gene annotation available. Upregulation of expression after vaccination was observed for 1879 loci, whereas 1022 loci showed a higher expression prior to vaccination.

**Figure 2:**
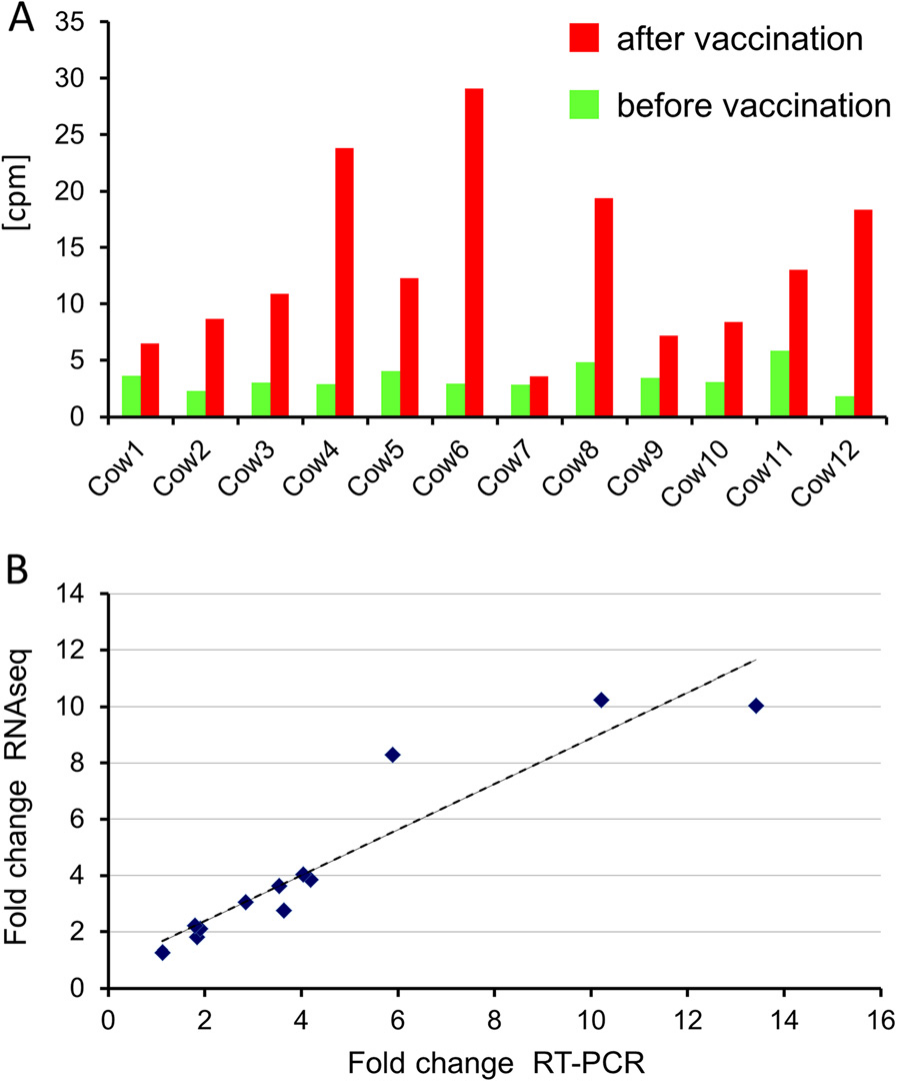
**Expression of the novel locus XLOC_032517 prior and after vaccination. A**: Number of transcripts for locus XLOC_032517 before and after vaccination in 12 cows determined by RNA seq. cpm: counts per million reads. **B**: Fold change at transcript level for locus XLOC_032517 after vaccination in 12 cows determined by RNAseq and single-locus quantitative RT-PCR.

**Figure 3:**
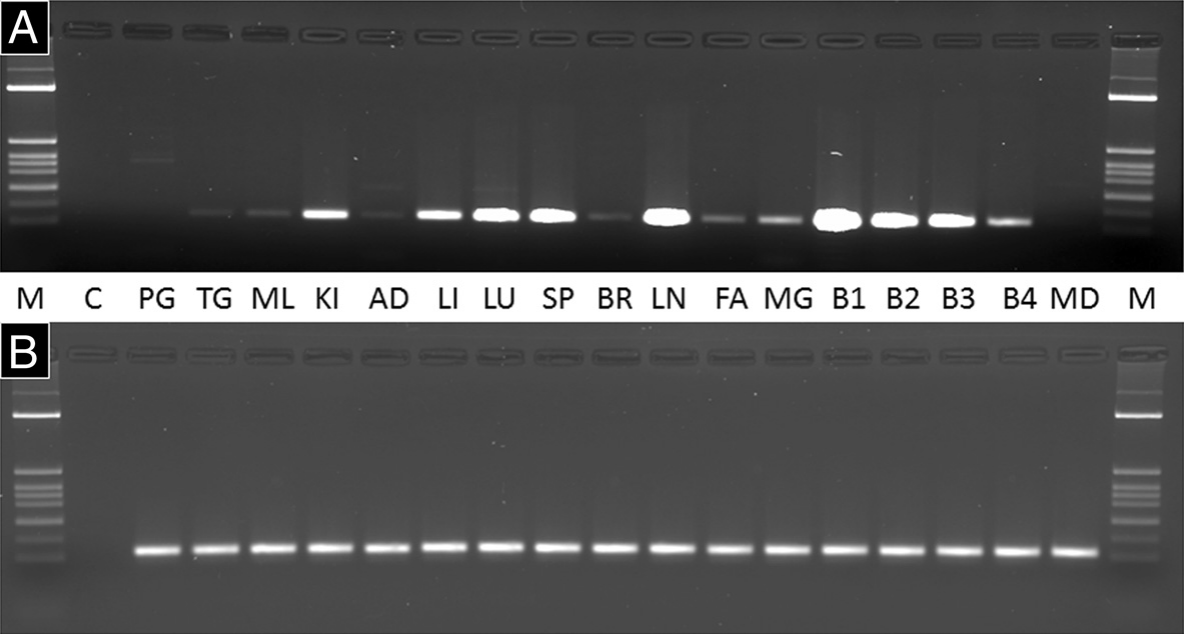
**Expression of XLOC_032517 in blood and other bovine tissues.** Detection of RT-PCR products from the XLOC_032517 locus **(A)** and the reference gene EIF3K **(B)** in cDNA from whole blood of cows, in bovine tissue samples and the MDBK cell line. M: Molecular weight marker, C: negative control without template, PG: pituitary gland, TG: thyroid gland, ML: skeletal muscle, KI: kidney, AD: adrenal gland, LI: liver, LU: lung, SP: spleen, BR: brain, LN: lymph node, FA: fat, MG: mammary gland, B1: blood sample from cow vaccinated with BVDV vaccine PregSure®, B2-B4: blood sample from cows vaccinated with an alternative BVDV vaccine, MD: MDBK cell line.

Our RNAseq analyses highlighted a strong upregulation of the previously unknown locus XLOC_032517 after vaccination in all samples (Figure 2A). In fact, XLOC_032517 is on top of the list (Additional file 2) of differentially expressed loci regarding statistical significance and second regarding fold change. In spite of the very strong regulation of XLOC_032517 expression after vaccination, there is no functional annotation of this locus neither in bovine nor in any other eukaryote genome. Locus specific RT-PCR analysis and subsequent sequencing of the obtained product approved the sequence of the transcript. Quantitative real-time PCR analysis confirmed a highly significant (*p* < 0.0001) 3.51 fold upregulation of XLOC_032517 expression after vaccination compared to the time point prior vaccination (Figure 2B). XLOC_032517 transcripts were also detected in blood samples of cows not vaccinated with the PregSure® vaccine (Figure 3). Three cows, which had never encountered PregSure® vaccination showed a significantly lower XLOC_032517 expression (*p* = 0.008) compared to the 12 cows, which had received at least three PregSure® vaccinations. Expression analyses of bovine tissues showed a strong XLOC_032517 expression in kidney, liver, lung, spleen and lymph node, a weak to moderate expression in thyroid gland, skeletal muscle, adrenal gland, brain, subcutaneous fat and mammary gland. No expression could be detected in the pituitary gland and in the MDBK cell line used for vaccine production.

**Figure 4:**
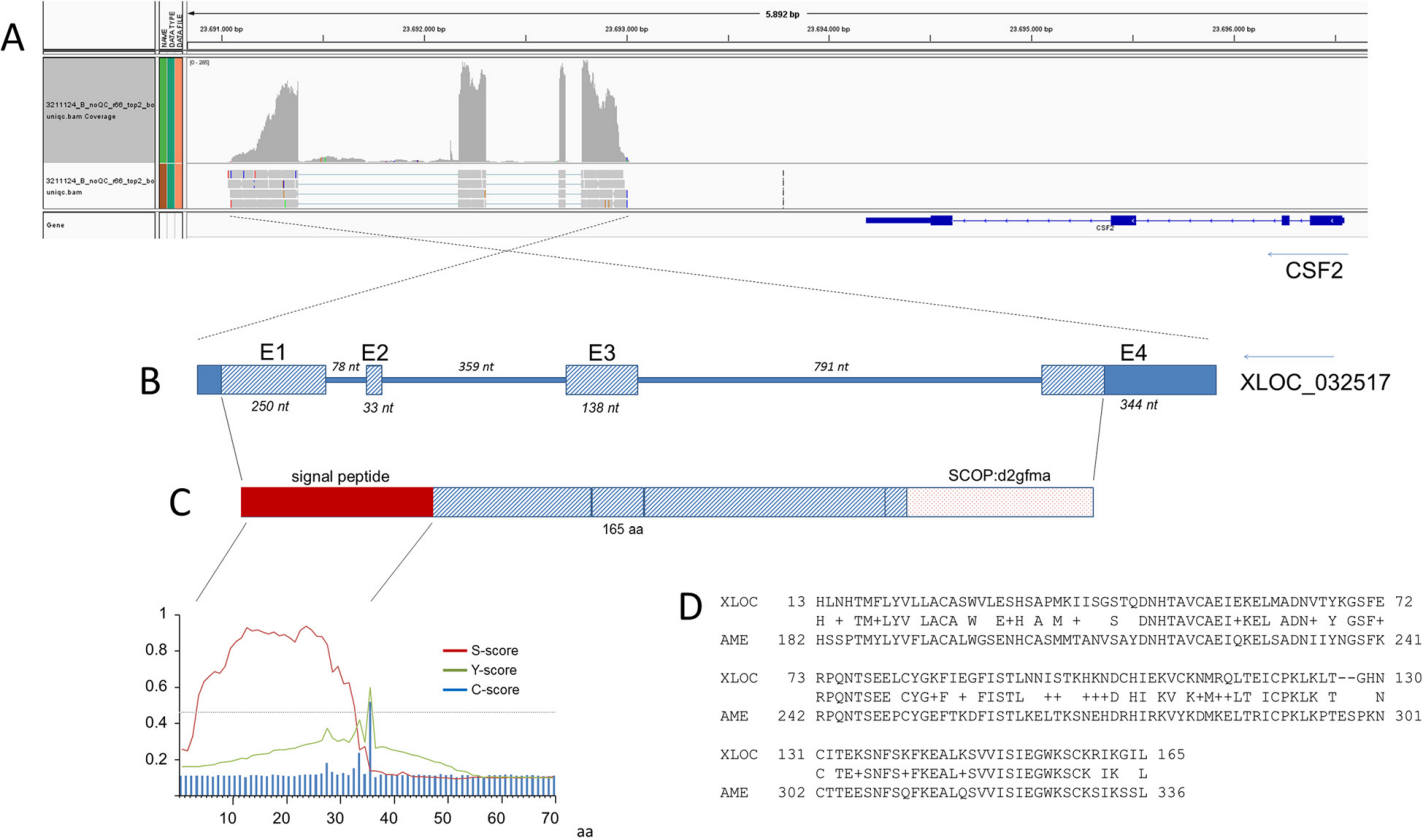
**Structural features of the novel bovine locus XLOC_032517. A**: Assignment of reads in the genomic region BTA7:23 691 000 Mb – 23 697 000 Mb visualised by Integrative Genome viewer. Annotation and orientation of the bovine CSF2 gene is indicated according to the NCBI Btau 6.1 assembly [33]. **B**: Genomic structure of XLOC_032517. Boxes indicate exons (E1-E4). Solid blue colour indicates the predicted 5’and 3’UTR, shaded pattern indicates an open reading frame with the predicted coding sequence. **C**: Structure of the predicted XLOC_032517 protein with signal peptide (red colour) and d2gfma motif (red dotted pattern). Significant evidence of the signal peptide motif is obtained by calculation of S-, Y-, and C-scores with SignalP [34]. **D**: Homology of the predicted XLOC_032517 amino acid sequence (XLOC) with the predicted great panda LOC100473502 amino acid sequence (AME) as determined by BLASTP [33].

The bovine locus XLOC_032517 showed a transcript length of 765 bp (Genbank Accession Nr. KF051797) and comprises 4 exons on chromosome 7 (Figure 4) in the region of 23.69 Mb between CSF2 and LOC789264. Analysis of a potential translation of the transcript sequence into amino acid sequence yielded an open reading frame from nt 48 to 542. Thus, the predicted bovine protein encoded by XLOC_032517 has 165 amino acids and a molecular weight of 18558.56 Daltons.

Due to lack of functional annotation for XLOC_032517 in public databases, bioinformatic analyses searched for similarity to sequences in other species to obtain information on the potential functional relevance of the locus. BLAST search at NCBI [33] with the XLOC_032517 sequence detected similar genomic sequences in a conserved exon-intron structure (essentially exons 1, 2, and 4) in the pig, dog, human and great panda genomes (Additional file 3). The sequence mapped *in silico* in an analogous chromosome interval flanked by the same neighbouring genes CSF2 and P4HA2. However, in those species there was also no functional annotation indicated for the respective chromosomal regions. Further genomic sequences with partial similarity to XLOC_032517 were found for sheep, lama, cat and horse (either from NCBI Refseq genomes or Whole Genome shotgun contigs deposited at NCBI). Searching the Ensembl data base [35] for cDNAs similar to XLOC_032517 identified no strong similarity to any known cDNAs in mammals. The CLTA-004 gene from human or great panda displayed only a weak similarity (< 70% across >130 bp) in this database. Further similarity search in the NCBI sequence repository brought forward five functionally non-annotated caprine sequences established by transcriptome shotgun assembly from blood (e.g., Accession Nr. JO333249.2). The identified caprine sequences showed a high similarity to XLOC_032517 (96% identity across 713 bp). BLASTP search for similar protein sequences revealed similarity only between the predicted amino acid sequence of the bovine XLOC_032517 and a hypothetical protein XP_002912997.1 of the LOC100473502 locus in the great panda, which had been predicted by bioinformatic analyses.

Missing functional annotation for sequences homologous to XLOC_032517 required further screening of the XLOC_032517 transcript for potential functionally relevant features. SignalP analysis [36] of the amino acid sequence of the predicted protein revealed a significant indication of a signal peptide cleavage site after amino acid (aa) 35 and a signal peptide region comprising aa 1 to 35 (see Figure 4). Furthermore, SMART analysis [37] indicated the additional features IL14/IL13 (aa 14 – 123) and CSF2 (aa 36 – 158). Although the CSF2 feature was below a significant threshold, the features IL14/IL13 and CSF2 would suggest a cytokine function of XLOC_032517, which fitted the prediction of a signal peptide for XLOC_032517 and also the feature SCOP: d2gmfa indicating a 4-helical cytokine [38,39]. Similarity to CSF2 is also supported by the genomic organisation of XLOC_032517, which is located adjacent to the CSF2 – IL3 cluster of colony stimulating factors on bovine chromosome 7 (BTA7). XLOC_032517 shares a similar four-exon structure with CSF2 and, like CSF2 and IL3, is placed in telomere – centromere orientation on the chromosome.

### Identification of differentially expressed annotated genes

Among the list of annotated differentially expressed loci in our experiment, many groups of genes with relevance in the immune response were highlighted. Specifically, interleukin signalling was affected indicated by differential expression of several interleukin receptor genes (IL18R1, IL21R, IL2RA, IL7R, and IL9R). Interleukin 1 (IL1) response is promoted after vaccination due to the upregulation of the IL1 receptor ligand IL1RL1 and the downregulation of IL1RN (receptor antagonist) and of IL1R2 (non-signalling receptor, q = 0.058). Furthermore, five members of the group of suppressors of cytokine signalling are upregulated after vaccination (SOCS2, SOCS4, SOCS5, SOCS6, SOCS7), and five ABC transporters (ABCB8, ABCB9, ABCB10, ABCD3, ABCE1) involved in the transport of molecules across extra- and intra-cellular membranes and particularly important in antigen presentation (ABCB8, ABCB9, [40]) were all upregulated. Upregulation was also observed for four genes encoding caspases (CASP3, CASP7, CASP8, CASP8AP2). Several chemokine receptors (CCR2, CCR4, CCR5, and CCR7) showed significantly differential expression as did inhibitors of nuclear factor of kappa light polypeptide gene enhancer in B cells (NFKBID, NFKBIL1, NFKBIZ). The three STAG genes (STAG1, STAG2, and STAG3), members of the cohesion complex [41], are all co-ordinately upregulated. In addition, structural maintenance of chromosome (SMC) condensins SMC2, SMC3, SMC4, SMC5, SMC6 and NCAPG, a non-structural maintenance of chromosomes condensin, are all higher expressed after vaccination compared to prior vaccination.

### Pathway analysis

Bioinformatic pathway analyses of the list of significantly differentially expressed genes (Additional file 2) after vaccination revealed a large number of significantly enriched pathways, biological functions and upstream regulators. Most of these pathways had a close association to immune response. Our data indicated a coordinated immune response to viral dsRNA sequences as well as an upregulation of T cell response and a response to an alloantigen.

#### KEGG pathways affected by vaccination

GOseq pathway analysis of differential expression revealed 29 significantly over-represented KEGG pathways (*p* < 0.05, Additional file 4). The *Ribosome* pathway stood out as the most significantly affected pathway. Furthermore, the list of the 10 most significantly enriched KEGG pathways also comprised *Allograft rejection*, *Graft-versus-host disease*, *Cytokine-cytokine receptor interaction*, *Natural killer cell mediated cytotoxicity*, and *RIG-I-like receptor signalling* indicating a major immune response to an alloantigen and a response to an RNA virus. Other significantly enriched KEGG pathways with an impact on immune response were the *MAPK signalling* pathway, the *T cell receptor signalling, the Toll-like receptor signalling, the Fc epsilon RI signalling* and *the antigen processing and presentation* pathway.

The KEGG pathway analysis confirmed that after vaccination the individuals responded to a viral RNA antigen due to overrepresentation of differentially expressed genes from the RIG-I-like receptor signalling pathway (Additional file 5) and upregulation of DDX58 encoding RIG I, a sensor of dsRNA [42]. Furthermore, the KEGG pathway downstream to the *RIG-I-like receptor signalling*, *MAPK signalling*, was confirmed as significantly affected by the vaccination in our transcriptome analysis. For the *MAPK signalling* pathway, differentially expressed genes of almost all parts of the pathway were observed.

#### Biological functions affected by vaccination

IPA analysis indicated that main biological functions affected by vaccination (Additional file 6) can be separated into four main groups: biological functions indicating an activation of gene expression, biological functions related to blood cell development and differentiation, biological functions involved in cell death, cell cycle and survival, and the biological function of ubiquination. Specifically, the functions *differentiation of blood cells*, d*evelopment of blood cells* and *development of leukocytes* or *quantity of T lymphocytes* (Additional file 6) showed a highly significant overrepresentation of genes. Other categories of biological functions affected by vaccination comprise *infectious disease*, *cell mediated immune response*, *humoral immune response*, *immunological disease*, c*ellular assembly and organisation*, c*ellular development*, c*ellular function and maintenance*, *haematological system development and function*, *lymphoid tissue structure and development, protein synthesis* and *posttranslational modification*.

#### Canonical pathways affected by vaccination

A total of 178 different canonical pathways were found significantly overrepresented as a response to vaccination (Additional file 7) in the IPA analysis. The list included many pathways known to be crucial during immune response. The canonical pathways highlight two key mechanisms in response to vaccination: response to dsRNA or a dsRNA analogue and upregulation of T cell receptor signalling.

In addition to the RIG I signalling pathway identified in KEGG, IPA showed a significant enrichment of differentially expressed loci for main mechanisms of protection against RNA viral infection: *EIF2 signalling* and TLR3 signalling within the *TLR signalling pathway*. Further pathways related to virus infection that are significantly enriched with differentially expressed loci are *NFkB activation by viruses* and *virus entry via endocytic pathways.*

The IPA analysis highlighted the *EIF2 signalling* (Additional file 8) as the most significantly enriched pathway seen as an effect of vaccination. In our data set, the EIF2AK2 gene encoding PKR and EIF2A encoding phosphorylate translation initiation factor EIF2 α are significantly upregulated after vaccination. Also several other kinases and translation initiation factors in the *EIF2 signalling* pathway showed a higher expression level after vaccination compared to the status prior vaccination.

Within the canonical pathway *Toll-like receptor signalling*, specifically the string starting with TLR3, is significantly overrepresented in the list of differentially expressed genes after vaccination (Additional file 9). The TLR3 receptor is a specific receptor for dsRNA [43]. The respective gene is upregulated after vaccination. Furthermore, several genes downstream in the *TLR signalling* pathway that lead to transcription of proinflammatory cytokines are also co-ordinately upregulated (IRAK4, TAB2, MAP3K7 encoding TAK1, MAPK8 encoding JNK1 and FOS encoding c-fos).

Besides response to dsRNA or a dsRNA analogue, the upregulation of *T cell receptor signaling* is a second major regulatory response to vaccination identified in our data set by Goseq analysis of the KEGG database (see above) as well as by IPA analysis of canonical pathways (Additional file 10). The CD3G gene encoding a member of the T cell receptor cluster as well as ZAP70 encoding a protein kinase activated by TCR clustering are both upregulated. In addition, all three major downstream signaling pathways of ZAP70 (NF-AT, Ras and also PKC) contain several significantly upregulated molecules. Examples are PPP3CA and PPP3CB encoding peptides belonging to the PP2B complex and the NFAT5 gene. Support for the hypothesis of a ZAP70 downstream regulation is also supported by the canonical pathways *role of NFAT in regulation of immune response* and *PKCʘ signaling in T lymphoctes* being significantly enriched with differentially expressed genes. Upregulation of T cell receptor signaling is also confirmed by the pathway *CD28 signaling in T helper cells* being significantly enriched with differentially expressed genes. Most of those genes are upregulated, only MHC class II DOB is downregulated.

In contrast to T cell activation, the IPA analysis indicated a downregulation of B cell activation 14 days after vaccination in our data: Most of the genes in the significantly affected canonical pathway *B cell development* are downregulated (Additional file 11), and key molecules at the start points of B cell receptor signal transduction are also downregulated. CD79A and CD79B act as signal transducers of the B cell receptor in B cells, and both showed a higher expression prior to vaccination compared to 14 days after vaccination. Not only the initial signal transducers CD79A/B, but also a co-stimulator for B cell receptor signalling, CD19, is downregulated.

A further signalling receptor required for B cell activation, CD40 showed a significantly lower expression after vaccination. Cell surface molecules of the entire process of B cell development from stem cell to plasma cell are all downregulated with the exception of IL7-R and CD80/CD86. However, the latter molecules also appear on the surface of non-B cell antigen presenting cells and are not specific to the B cell lineage.

The canonical pathway *NFκB signalling* is significantly enriched for differentially expressed genes after vaccination. Specifically genes in the signalling cascade of NFκB for B cell maturation are co-ordinately downregulated (Additional file 12) adding further evidence towards suppression of B cell maturation.

#### Major upstream regulators

Pathway analysis identified major upstream regulators based on expected causal effects between upstream regulators and target genes and predicts activation or inhibition of those regulators. Highly significantly affected upstream regulators in our data set were v-myc myelocytomatosis viral related oncogene (neuroblastoma derived, MYCN), the T cell receptor complex TCR, the CD40 ligand (CD40LG), CD28, E2F transcription factor 1 (E2F1), interleukin 2 (IL2), transforming growth factor beta 1 (TGFB1), CD3, and microRNAs miR-30c-5p, miR-155-5p and miR-124-3p (Table 2, Additional file 13).

**Table 2:** List of the 20 most significant upstream regulators identified as involved in gene regulation after vaccination as determined by IPA analysis.

**Upstream regulator**	**Molecule type**	**Predicted activation state**^**1**^	***p*-value of overlap**	**Number of target molecules in dataset**
miR-30c-5p (and other miRNAs w/seed GUAAACA)	mature microRNA	Inhibited	1.75E-07	29
MYCN	transcription regulator	Inhibited	4.08E-07	52
TCR	complex		1.02E-06	57
miR-155-5p (miRNAs w/seed UAAUGCU)	mature microRNA	Inhibited	1.97E-06	34
CD40LG	cytokine	Activated	2.06E-06	67
CD28	transmembrane receptor	Inhibited	6.13E-06	62
mir-210	microRNA		1.58E-05	16
E2F1	transcription regulator	Activated	8.88E-05	52
miR-124-3p (and other miRNAs w/seed AAGGCAC)	mature microRNA	Inhibited	1.23E-04	38
HOXA9	transcription regulator		1.33E-04	34
IL2	cytokine	Activated	1.77E-04	58
CD3	complex	Inhibited	4.26E-04	98
miR-291a-3p (and other miRNAs w/seed AAGUGCU)	mature microRNA	Inhibited	4.55E-04	18
SMARCC1	transcription regulator		7.17E-04	4
YWHAQ	other		7.37E-04	5
SHOX	transcription regulator		7.37E-04	5
TGFB1	growth factor	Activated	7.99E-04	187
FSH	complex		1.64E-03	60
TMBIM6	other		1.68E-03	6
miR-17-5p (and other miRNAs w/seed AAAGUGC)	mature microRNA	Inhibited	1.94E-03	10

^1^ For identification of activation or inhibition of upstream regulators, a threshold for the activation z-score calculated in the IPA analysis of |z-score| > 2 was applied.

## Discussion

Our study investigated the recall response to vaccination with the vaccine associated with BNP. The potential relevance of a recall response to this vaccine for BNP is underlined by data indicating that an increased frequency of vaccination in an individual is associated with increased alloantibody titres [8].

### Identification of a novel locus significantly related to immune response

Applying the holistic deep sequencing approach to the entire bovine blood transcriptome, we identified 4596 previously unknown transcribed loci in the bovine genome. This confirms the power of the RNAseq approach to reveal novel functional elements in immune response. A prime example for the potential relevance of these new functional elements is the gene XLOC_032517, a previously unknown locus with the most significant difference of all investigated loci regarding expression related to vaccination with a specific BVDV vaccine. The new gene could be detected in bovine blood samples of divergent origin and also in a variety of tissues except pituitary gland. The level of expression varied between tissues with those tissues showing the strongest expression, which have a particular relevance during immune response to invading pathogens (e.g. lymph node, spleen, and lung).

There is no annotation of the locus in any other species, and also no transcripts with homology to XLOC_032517 were identified in any database except for goat. This might indicate that the XLOC_032517 is specific to ruminant immune response, although a predicted protein from *Ailuropoda melanoleuca* (XP_002912951.1) has a partial similarity to the predicted XLOC_032517 protein. The partially conserved exon-intron-structure of homologous sequences in the human and other genomes might point forward to an evolutionary process for XLOC_032517 to become transcriptionally active or silenced, respectively.

Due to lack of any previous data on XLOC_32517, its functional role was completely unknown. Bioinformatic search for functionally relevant structural features of locus XLOC_032517 added further pieces of evidence that the new gene is involved in immune response due to its similarity with structural features identified in CSF2 and IL14/IL13 and its predicted affiliation to the 4-helical cytokine superfamily. Studies from human indicate that CSF2 is expressed in human leukocytes especially upon activation [44]. Interestingly, only for CSF1, expression could be detected in our data set, whereas no expression of the other genes encoding colony stimulating factors (CSF2, CSF3 or IL3) was observed. This pattern is in agreement with the data set from O’Loughlin et al. [45], who performed an RNAseq experiment on calf leukocytes before and after weaning. It has to be evaluated, if XLOC_032517 is a bovine functional surrogate to CSF2. CSF2 is not expressed in bovine blood cells in our data set, although there is a significant and coordinated upregulation after vaccination of several genes encoding proteins of a specific transcription factor complex (calcineurinA-calcineurinB-Runx1: PPP3CA, PPP3CB, RUNX1) relevant for up-regulation of CSF2 [46]. In summary, the *in silico* analyses advocate that the predicted protein encoded by XLOC_032517 might be a new member of the colony stimulating factor family in cattle. Further studies will have to confirm the predicted protein coding potential of XLOC_032517 and the expression pattern in different specific leukocyte populations.

### Immune response to dsRNA or a dsRNA analogue after vaccination

**Figure 5:**
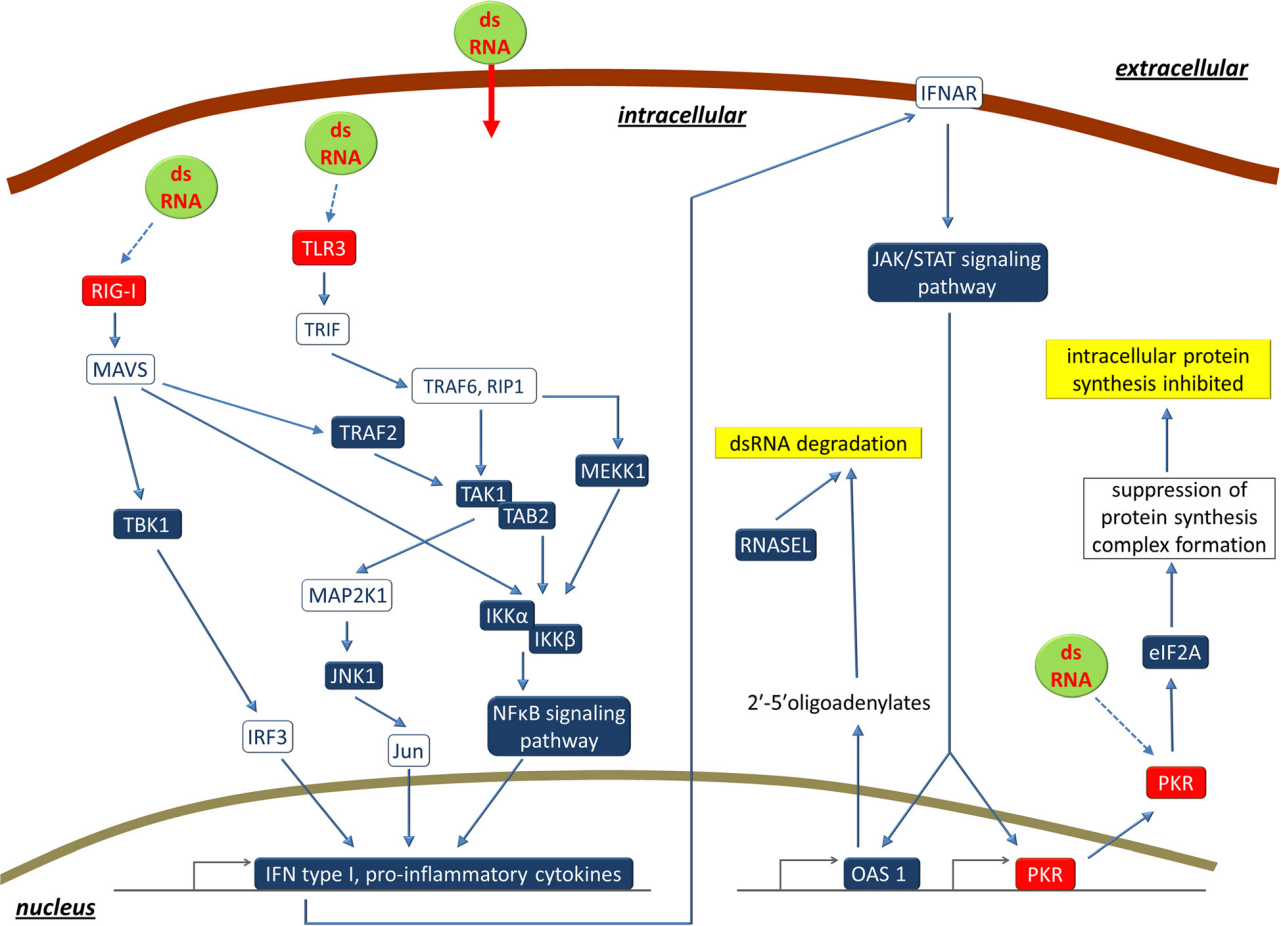
**Model for the coordinated response to dsRNA after vaccination with the inactivated vaccine PregSure® against ssRNA BVD virus.** Coloured boxes indicate differentially expressed genes or significantly affected pathways when comparing gene expression before and after vaccination. Red boxes indicate intracellular sensors of dsRNA; blue boxes indicate other genes or pathways. Red arrow indicates transport of dsRNA into the cell, the blue dotted lines indicate activation of dsRNA sensors by dsRNA, and the blue solid lines indicate other regulatory or activating effects.

The pathway analysis of the expression data from whole blood transcriptomes highlighted a very specific immune response to viral dsRNA or a dsRNA analogue after vaccination (Figure 5). KEGG as well as IPA pathway analyses confirmed that the individuals responded to virus RNA demonstrated by overrepresentation of differentially expressed genes from key pathways of innate recognition of viruses: the *RIG-I-like receptor signalling* pathway, the *TLR/TLR3 signalling* pathway, and the *EIF2 signalling via PKR*[47].

The *EIF2 signalling* pathway is the most significantly vaccination-affected canonical pathway according to IPA analysis. *EIF2 signalling* is affected by dsRNA-viral infection via PKR. The endpoint of the *EIF2 signalling* pathway is the regulation of translation. The EIF2AK2 gene encoding PKR and EIF2A encoding EIF2 α are both upregulated after vaccination. After viral contact the PKR is activated by autophosphorylation after binding to dsRNA [47]. The activated form of the PKR can phosphorylate translation initiation factor EIF2 α, which in turn inhibits protein synthesis via suppression of protein synthesis complex formation. Downregulation of protein translation is a method of protecting the individual against virus replication by preventing viral structural protein synthesis [48]. A downregulation of translation is also confirmed by analysis of biological functions affected by vaccination (Additional file 6). Phosphorylation of EIF2A via PRK has also been shown to mediate apoptosis by increasing caspase 3 [49], which is in agreement with the CASP3 upregulation after vaccination in our data set. Further support for the hypothesis of a downregulated protein synthesis after vaccination is obtained from *Ribosome* being by far the most significantly affected KEGG pathway after vaccination (Additional file 4). Specific analysis of the respective genes in that pathway indicated that the vast majority of ribosomal protein encoding genes, which showed a significantly differential expression after vaccination, was all concordantly downregulated (e.g., 45 of the total of 46 differentially expressed RPL and RPS genes, Additional file 2).

The second mechanism significantly affected by vaccination, which protects the individual specifically against RNA viral infection, is the RIG I signalling pathway (Figure 5) [42,50]. The gene DDX58 encoding RIG I is upregulated (*p* = 0.058). RIG I acts as a cytoplasmic sensor of viral nucleic acids [42] and induces pro-inflammatory cytokine and interferon I expression. Posttranslational polyubiquination of RIG I is pivotal for activation of RIG I. We found increased ubiquitination of proteins in the list of significantly affected biological functions (see Additional file 6) after vaccination. Activated ubiquitination might thus be relevant in our experiment for RNA sensor signalling in addition to its known role during proteasomal degradation, e.g. of viral peptides for MHC class I presentation [51].

The third mechanism of protection against viral dsRNA (Figure 5) identified in response to vaccination in our dataset is the activation of the TLR3 signalling pathway known to upregulate the synthesis of proinflammatory cytokines after TLR3 activation by dsRNA [43,47].

The cytokine activation might relate to a further machinery of protection against flaviviral invasion that is upregulated after vaccination: RNA degradation (Figure 5). The OAS1 gene is upregulated in our data set. OAS1 is induced by α interferons and activates RNASE L by synthesising 2’,5’-oligoadenylates (2-5As) [48]. RNASE L degrades viral RNA and inhibits viral replication. This mechanism can be induced by interferon type I signalling: a respective upregulation of IFNA16, member of the antiviral IFNα family, was detected in our data set. Alternatively, OAS1 is able to act as sensor for dsRNA directly. Interestingly, those two Interferon-type I-induced antiviral pathways that also act by direct recognition of dsRNA (OAS1, PKR, [48]) are significantly affected by BVDV vaccination in our data set. In contrast, those other interferon-type I-induced antiviral pathways (MX1, ADAR) not affected by vaccination, lack dsRNA sensor activity and are exclusively dependent on IFN alpha signalling [48].

Obviously the upregulation of the protection against dsRNA is no short term effect, because significantly differential expression could be detected 14 days after application of the vaccine suggesting a long lasting release of dsRNA or dsRNA analogue molecules from the injection site. The differential expression of the dsRNA sensors PKR, TLR3, and DDX58 (Figure 5) and the affected downstream pathways in response to vaccination implicate that the cells monitored in the blood must have had contact with dsRNA. One explanation might be the roaming of cells that incorporated viral dsRNA at the injection site. However, PregSure® is an inactivated vaccine against the BVD virus, an ssRNA pestivirus belonging to the Flaviviridae [52]. Thus, the vaccine should only contain ssRNA. This raises the question as to the origin of the coordinated viral-dsRNA-sensor upregulation in blood cells after vaccination. Due to the inactivated virus in the vaccine, intracellular virus replication after infection known to generate dsRNA by-products [47] should be excluded. Thus, the vaccine itself must have contained dsRNA or a dsRNA analogue. The dsRNA or the analogue could either originate from cellular remnants of the host cells after virus cultivation for vaccine production or were intentionally added to the vaccine as adjuvant. Vaccine contamination fits the hypothesis of a massive contamination of the PregSure® vaccine with components of the MDBK host cells, a bovine kidney epithelial cell line, as indicated by [53]. This massive vaccine contamination with bovine cellular contaminants could also be responsible for the massive production of alloantibodies in cows producing BNP inducing colostrum. Alternatively, the vaccine might have been modulated by addition of a dsRNA analogue, e.g. polyinosinic:polycytidylic acid (poly I:C), a toll-like receptor (TLR) agonist that mimics the immunostimulatory properties of dsRNA, which has been suggested to improve vaccination effects (e.g., [54]).

### Coordinated regulation of groups of genes relevant for immune response

In addition to the coordinated activation of dsRNA response mechanisms, the concordant differential expression of several members from groups of immune - relevant genes further indicated a coordinated regulation of a variety of immune response components to vaccination. The promoted interleukin 1 (IL1) response is one example, It is supported by the differentially expressed nuclear factor of kappa light polypeptide gene enhancer in B cells inhibitors (NFKBID, NFKBIL1, NFKBIZ) that are known to be involved in the induction of inflammatory genes activated through TLR/IL1 signalling [55].

Another example for the concordant regulation of several genes from one immune-relevant family is the upregulation of SOCS genes, which might represent a negative feedback to interleukin receptor upregulation according to SOCS’ known inhibitory effects on cytokine signalling [56].

Furthermore, upregulation of members of the cohesion complex and also SMC and non-SMC condensins that are essential during mitosis suggest a promotion of cell division.

### T cell and B cell response

In our data set, we observed a coordinated upregulation of T cell receptor signalling upon vaccination. Signal transduction goes via CD3, and subsequently ZAP70, which are both upregulated. ZAP70 is known to be involved in protein kinase C θ and calcineurin-induced transcriptional activity [57]. Three major downstream signalling pathways of ZAP70 (NF-AT, NFκB and also JUN/FOS, [58-60]) contain several significantly upregulated molecules. For example, in the *NF-AT signalling* pathway, PPP3CA and PPP3CB belonging to the PP2B complex are upregulated as well as NFAT5, encoding a factor of activated T cells important for induction of gene expression: the NFAT5 protein belongs to the group of nuclear factors that are crucial for inducible gene transcription during immune response [61].

There is a coordinated regulation between genes presumably acting in antigen presenting cells and genes presumably acting in the T cell populations: the gene CD3G encoding a protein within the T cell receptor is upregulated after vaccination. The CD80 and CD86 genes encoding proteins on the surface of antigen presenting cells also show a higher expression after vaccination. The interaction of CD80 with ligands, e.g. CD28 or CD152 is important for T cell communication with the antigen-presenting cells [62]. CD28 is highlighted as a major upstream regulator in IPA analyses. In addition, the MHC class II gene DO also expressed in antigen presenting cells is downregulated after vaccination. In humans, the DO heterodimer expressed in antigen presenting cells (macrophages, dendritic cells) suppresses peptide loading of MHC class II molecules by inhibiting HLA-DM [63]. Upregulation of CD80 and downregulation of Bola DOB in our data set indicates an activation of antigen presentation in the antigen presenting cells after vaccination.

A potential effector mechanism after T cell activation is the induction of apoptosis in the antigen presenting cells via caspase: initiator caspase CASP8 and its interacting protein CASPA8AP2 as well as the effector caspases CASP3 and CASP7 are all upregulated. The hypothesis of induced apoptosis is also supported by the significantly affected canonical pathway *mitochondrial dysfunction* suggesting activation of the intrinsic or mitochondrial pathway of apoptosis and by the increased expression of a member of the tumor necrosis factor (ligand) superfamily, TNFSF10 (TRAIL). TRAIL has been shown to trigger the activation of MAPK8/JNK, CASP8, and CASP3 [64].

The hypothesis of T cell induced apoptosis fits also the upregulation of GZMA expression encoding granzyme A after vaccination. Granzyme A synthesis and transmission to target cells is a mechanism of cytotoxic T cells killing antigen presenting target cells via the intrinsic apoptosis pathway [65]. In addition to cytotoxic T cells, granzyme is also produced by Natural killer (NK) cells. These cells might also contribute to the elevated transcript level of the GZMA gene, because there is a significant upregulation of genes encoding NK cell lectin-like receptors [KLRK1 and LOC100294723 (Killer cell lectin-like receptor subfamily B member 1B allele A-like)]. Interestingly, NKG2 encoded by KLRK1 recognizes non-classical MHC class I proteins like polymorphic MICA and MICB produced by stressed cells [66]. MICA is a stress-induced self-antigen frequently expressed in virus-infected cells. This result adds further indicators for a specific immune response to virus after vaccination with the BVDV vaccine.

In contrast to T cell activation, surprisingly B cell activation 14 days after vaccination with the BVDV vaccine seems to be suppressed. In addition to signal transducing peptides CD79A, CD79B and CD19, also cell surface molecules along the entire process of B cell development from stem cell to plasma cell are downregulated (IgM, IgD, Mu). Deactivation of B cells is also supported by the downregulation detected for TNFRSF13C. TNFRSF13C is a receptor for B cell-activating factor (BAFF), which is known to enhance B-cell survival in vitro and is a regulator of the peripheral B cell population [67]. The indication on suppression of B cell response at transcriptome level 14 days after vaccination is particularly unexpected, because the vaccine is known for high production of neutralising BVD antibodies [14]. The reason for the lack of B cell activation at this time point is unknown. It might be speculated that there is a negative feedback at the level of transcriptional regulation to counteract a high B cell response prior to day 14 post vaccination.

### Alloimmune response

KEGG pathway analysis indicated the *allograft rejection* pathway as significantly enriched with differentially expressed genes. This would fit the hypothesis of a substantial immune response to an alloantigen from the BVDV vaccine [3-5]. However, close examination of the expression of key mediators of alloantigen response indicated that there was no coordinated pattern of expression indicating an activation of this pathway. On the one hand, CD80 and CD86 expressed on professional antigen presenting cells (e.g., macrophages) are both upregulated and MHC class II DO (inhibitor of antigen binding to MHC, see above) is downregulated. This would suggest an activation of alloantigen response. On the other hand, CD40 was downregulated, but for CD40LG, one of the major upstream regulators identified in our data set (see Table 2), activation was predicted. CD40 is relevant for B cell activation [68], which is important for antibody mediated alloantigen response and also for macrophage mediated cytotoxic alloantigen response.

### Comparison to previous studies

Comparing our data with a previous study applying deep transcriptome sequencing in cattle [12] on the immune response to vaccination against Mycobacterium bovis showed that there was only a limited overlap of affected pathways between that study and our data. Only the *cytokine – cytokine receptor interaction* pathway was significantly overrepresented with differentially expressed genes in both studies. However, it has to be considered that Bhuju and colleagues [12] used a very different kind of vaccine directed against an intracellular bacterial pathogen, investigated specifically PBMC leukocyte expression and used a bovine genome annotation restricted to genes in the previous Btau4 assembly. In contrast, our study monitored the effect of an inactivated ssRNA virus vaccine in whole blood samples immediately stored after collection without any preparatory manipulation. Vartanian and colleagues [31] summarised the advantages of analysing whole blood compared to specific cell fractions. In addition, we specifically generated a comprehensive annotation of the bovine genome for our analysis. We did not find any equivalent further studies of vaccination effects of inactivated viral vaccines with deep sequencing techniques of blood transcriptomes in mammals. Whereas there are multiple studies on immune response to anti-viral vaccination at transcriptome level using microarrays in human, chicken, pigs or aquaculture (e.g. [69]), to the author’s knowledge respective data are missing in cattle for vaccinations similar to our regime.

### Conclusions

RNAseq of blood samples is restricted to the analysis of those transcript-carrying cells roaming in the blood stream. Any cellular regulation involved in immune response restricted to the periphery is not captured by this technique. However, our analyses demonstrated that a comprehensive regulation of the immune response to vaccination can be detected by our approach.

Our data showed a very coordinated immune response to dsRNA or a dsRNA analogue after vaccination with the specific, inactivated BVDV vaccine associated to BNP. This indicates a massive contamination of the vaccine, because the vaccine is inactivated and directed against BVDV, an ssRNA virus. Alternatively, a dsRNA mimicking agent, e.g., polyinosinic:polycytidylic acid (poly I:C), a toll-like receptor (TLR) agonist might be constituent of the vaccine adjuvant. The vaccine used in our experiment had been demonstrated to generate BNP-associated alloantibodies, which are probably directed against cellular contaminations of the vaccine. A contamination of the vaccine by MDBK host cell proteins has been demonstrated [53]. However, our data did not indicate a major, coordinated response to an alloantigen after vaccination. Thus, in contrast to the immune response to dsRNA or an analogue, there is no clear mechanism identified for activated alloantigen response after vaccination with the BVDV vaccine from our data set. However, we did observe a previously unknown locus highly upregulated after vaccination with potential cytokine features. It will have to be investigated in future studies, if this novel gene is specifically related to the vaccine used in this experiment and also to BNP or if it is a gene with a general function in immune response.

## Competing interests

The authors declare that they have no competing interests.

## Authors’ contributions

WD carried out the experiment, contributed to data collection and analysis, and participated in drafting the manuscript; RW participated in the design of the study, conceived the qRT-PCR experiments and participated in drafting the manuscript; FH participated in data analysis and drafting the manuscript; KM participated in conceiving the study and drafting the manuscript, and CK participated in conducting the experiment, carried out the data analysis, conceived the experiment and prepared the manuscript. All authors read and approved the final manuscript.

## Supplementary Material

Additional file 1 **List of primers used for genomic confirmation and differential expression of the novel locus XLOC_032517.** The table lists the sequences of all primers used for confirmation of structure and differential expression of the new locus XLOC_032517. Click here for file

Additional file 2 **List of differentially expressed loci prior vs. after vaccination at a threshold of q < 0.1.** The table provides a comprehensive list of all loci showing differential expression in response to vaccination at a significance threshold of q < 0.10. LogFC: log fold change between expression levels prior and after vaccination, logCPM: log cpm; gene: official locus name or previously unknown locus (= no annotat.), locus: chromosomal position of the expressed locus. Click here for file

Additional file 3 **Conservation of exon-intron structure in the human genome for a sequence homologous to XLOC_032517.** The figure provides the result from a BLAST search with the XLOC_032517 transcript (Genbank Accession No. KF051797) against all human genome assemblies deposited at NCBI. Click here for file

Additional file 4 **List of significantly overrepresented KEGG pathways established by GOseq analysis from the list of significantly differentially expressed genes after vaccination.** The table lists all significantly affected KEGG pathways with the respective p-values for overrepresentation of genes showing a significantly different expression in response to vaccination. Click here for file

Additional file 5 **KEGG pathway RIG I like receptor signalling.** The overview of the KEGG RIG I like receptor signalling pathway indicates significantly affected downstream KEGG pathways and differentially expressed genes after vaccination with the PregSure® vaccine obtained from GOseq analyses. All non-differentially expressed genes have a white background, all upregulated genes have an orange-red background and all downregulated genes have a green background. All differentially expressed genes or significantly affected pathways share the red frame. Solid red frames indicate results obtained from the KEGG data base, dashed red frames highlight significantly affected biological functions as indicated by IPA analysis. Click here for file

Additional file 6 **List of biological functions significantly affected by vaccination as determined by IPA analysis.** The folder “categories of affected function” summarises the affected biological functions into categories according to IPA analysis. Furthermore, the range of p values for overrepresentation and the number of differentially expressed loci in each category is indicated. The folder “affected_biological_functions” lists the individual biological categories with a significant overrepresentation of differentially expressed genes. Furthermore, the predicted activation status and the number of differentially expressed genes assigned to the respective function are indicated. Click here for file

Additional file 7 **List of canonical pathways significantly affected by vaccination as determined by IPA analysis.** The table lists all significantly affected canonical pathways obtained by IPA analysis with the respective p-values for overrepresentation of genes showing a significantly different expression in response to vaccination. The ratio provides the proportion of genes in the respective canonical pathway, which are significantly differentially expressed after vaccination, relative to all genes in the pathway. In addition, the proportion of upregulated and downregulated genes in the respective pathway and a full list of all differentially expressed genes assigned to the respective pathway are indicated. Click here for file

Additional file 8 **EIF2 signalling canonical pathway significantly affected by vaccination.** The figure highlights all elements within the IPA canonical pathway EIF2 signalling differentially expressed after vaccination. Orange/red elements: upregulated after vaccination, green elements: downregulated after vaccination. Colour intensity reflects the different fold change of expression. Click here for file

Additional file 9 **TLR signalling canonical pathway significantly affected by vaccination.** Elements within the IPA canonical pathway TLR signalling differentially expressed after vaccination are indicated. Orange/red elements: upregulated after vaccination, green elements: downregulated after vaccination. Colour intensity reflects the different fold change of expression. Click here for file

Additional file 10 **T cell receptor signalling canonical pathway significantly affected by vaccination.** Elements within the IPA canonical pathway T cell receptor signalling differentially expressed after vaccination are indicated. Orange/red elements: upregulated after vaccination, green elements: downregulated after vaccination. Colour intensity reflects the different fold change of expression. Click here for file

Additional file 11 **B cell development canonical pathway significantly affected by vaccination.** Elements within the IPA canonical pathway B cell development differentially expressed after vaccination are indicated. Orange/red elements: upregulated after vaccination, green elements: downregulated after vaccination. Colour intensity reflects the different fold change of expression. Click here for file

Additional file 12 **NFκB signalling canonical pathway significantly affected by vaccination.** Elements within the IPA canonical pathway NFκB signalling differentially expressed after vaccination are indicated. Orange/red elements: upregulated after vaccination, green elements: downregulated after vaccination. Colour intensity reflects the different fold change of expression. Click here for file

Additional file 13 **List of target molecules for predicted upstream regulators of differential expression after vaccination as determined by IPA analysis.** The file provides a list of predicted upstream regulators obtained by IPA analysis from the data set of differentially expressed genes. In addition, for all upstream regulators, all respective differentially expressed target genes are indicated. Click here for file
