# Supplementary figures and images for: Monitoring the immune response to vaccination with an inactivated vaccine associated to bovine neonatal pancytopenia by deep sequencing transcriptome analysis in cattle
Source: Vet Res. 2013 Oct 7;44(1):93. doi: 10.1186/1297-9716-44-93 (PMC3851820; doi:10.1186/1297-9716-44-93)

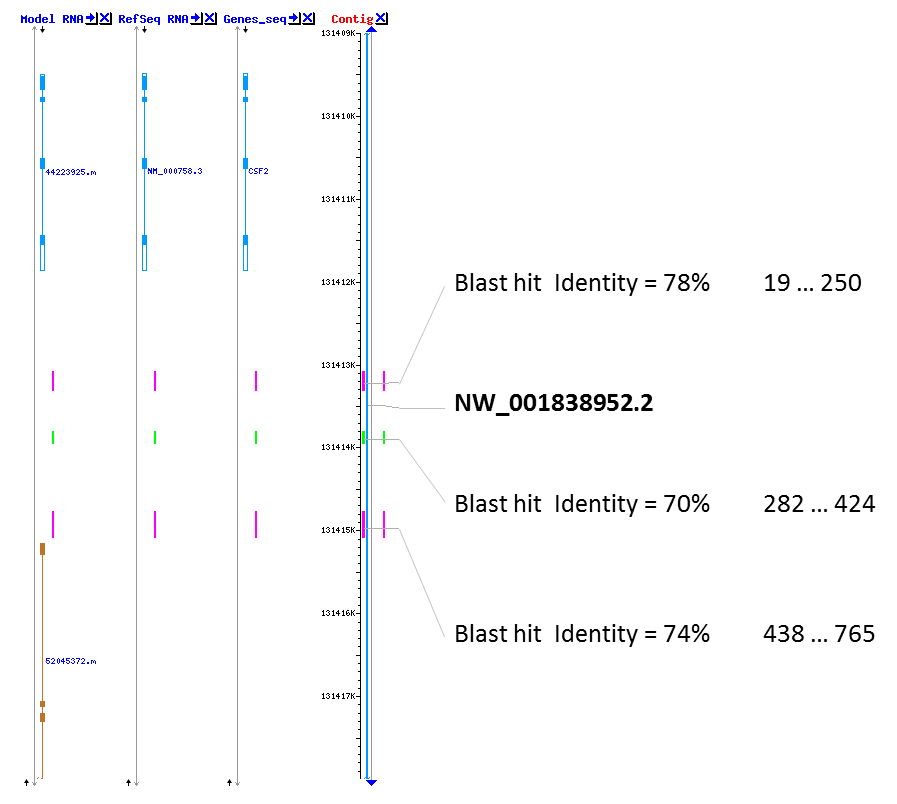

Supplement: Additional file 3 — Conservation of exon-intron structure in the human genome for a sequence homologous to XLOC_032517. The figure provides the result from a BLAST search with the XLOC_032517 transcript (Genbank Accession No. KF051797) against all human genome assemblies deposited at NCBI. [file 1297-9716-44-93-S3.tiff]

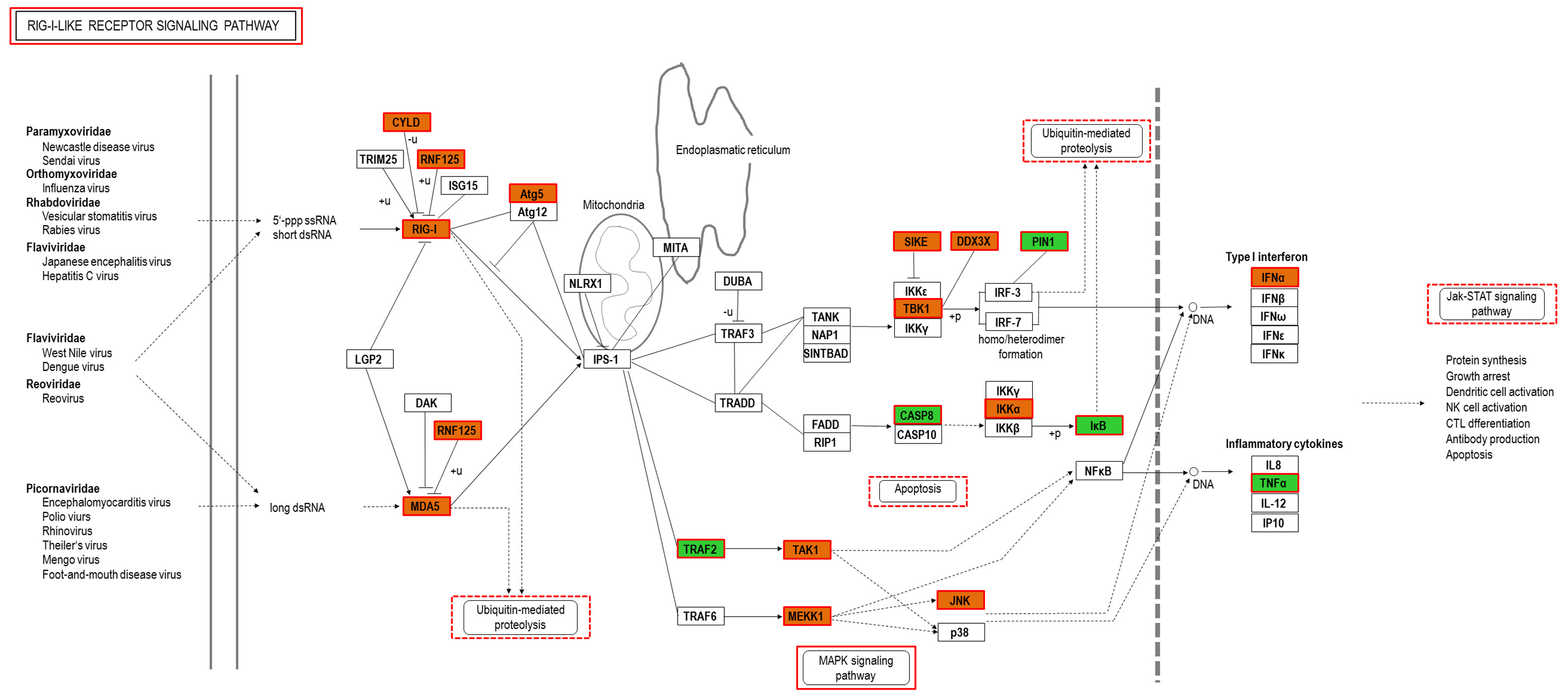

Supplement: Additional file 5 — KEGG pathway RIG I like receptor signalling. The overview of the KEGG RIG I like receptor signalling pathway indicates significantly affected downstream KEGG pathways and differentially expressed genes after vaccination with the PregSure® vaccine obtained from GOseq analyses. All non-differentially expressed genes have a white background, all upregulated genes have an orange-red background and all downregulated genes have a green background. All differentially expressed genes or significantly affected pathways share the red frame. Solid red frames indicate results obtained from the KEGG data base, dashed red frames highlight significantly affected biological functions as indicated by IPA analysis. [file 1297-9716-44-93-S5.jpeg]

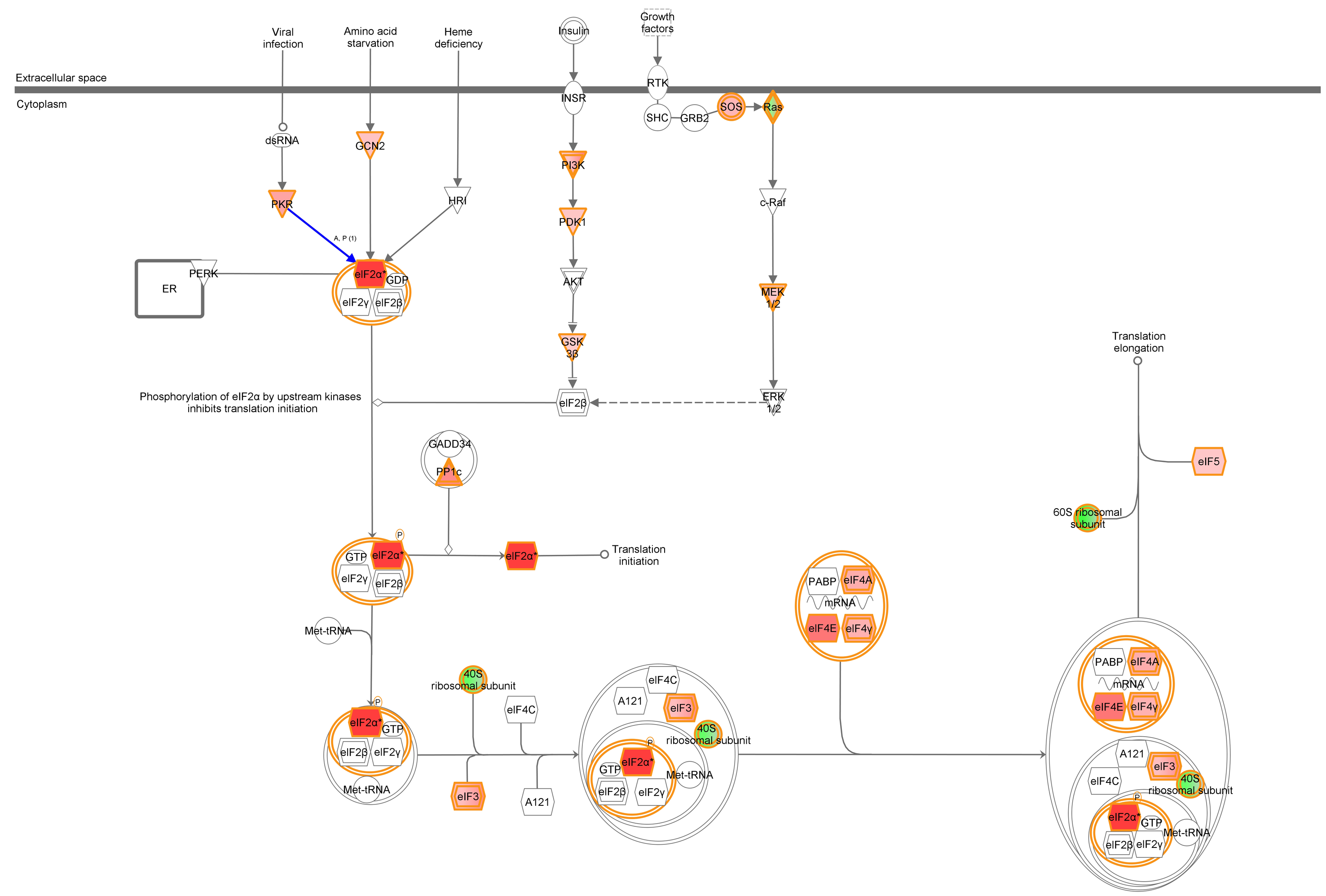

Supplement: Additional file 8 — EIF2 signalling canonical pathway significantly affected by vaccination. The figure highlights all elements within the IPA canonical pathway EIF2 signalling differentially expressed after vaccination. Orange/red elements: upregulated after vaccination, green elements: downregulated after vaccination. Colour intensity reflects the different fold change of expression. [file 1297-9716-44-93-S8.tiff]

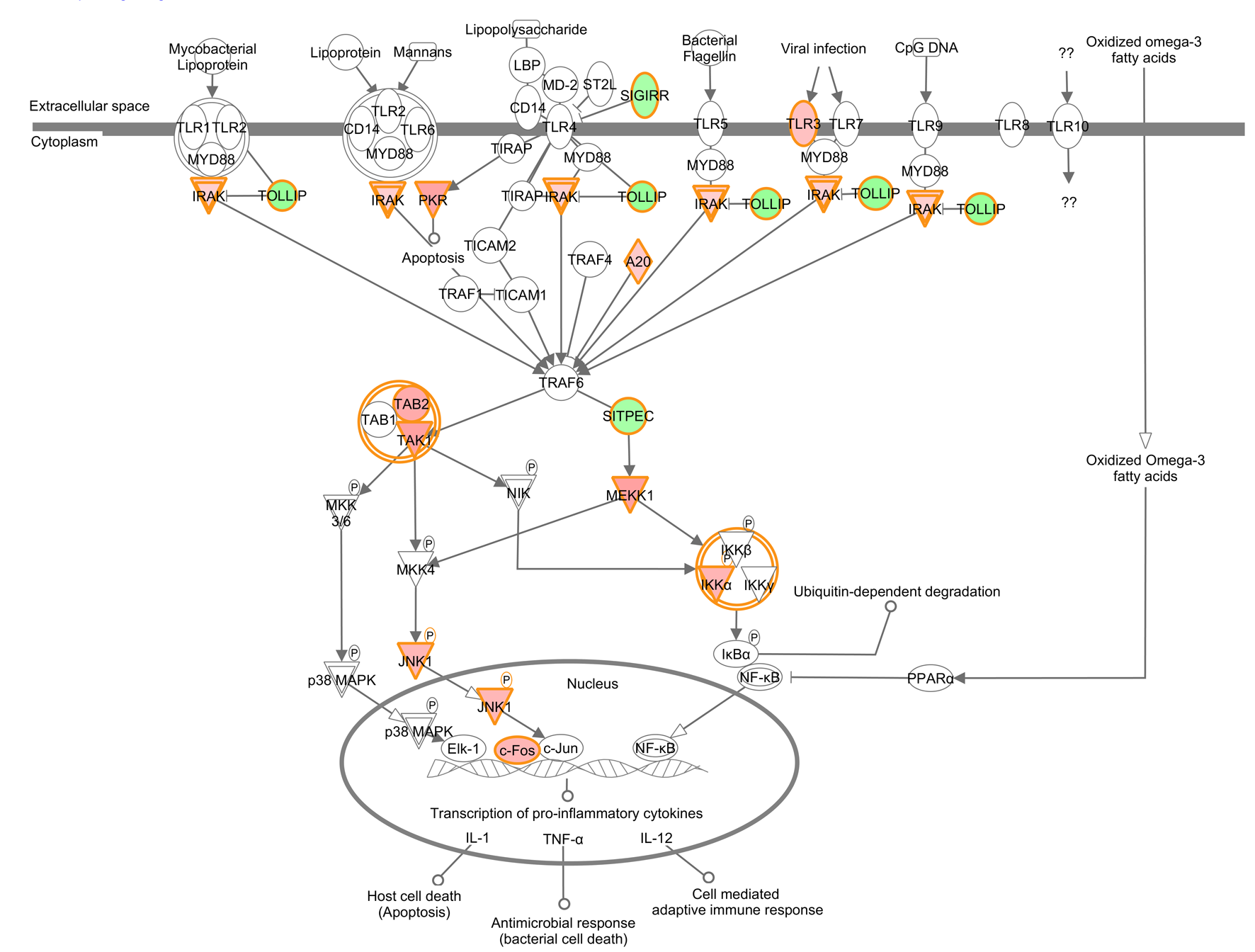

Supplement: Additional file 9 — TLR signalling canonical pathway significantly affected by vaccination. Elements within the IPA canonical pathway TLR signalling differentially expressed after vaccination are indicated. Orange/red elements: upregulated after vaccination, green elements: downregulated after vaccination. Colour intensity reflects the different fold change of expression. [file 1297-9716-44-93-S9.tiff]

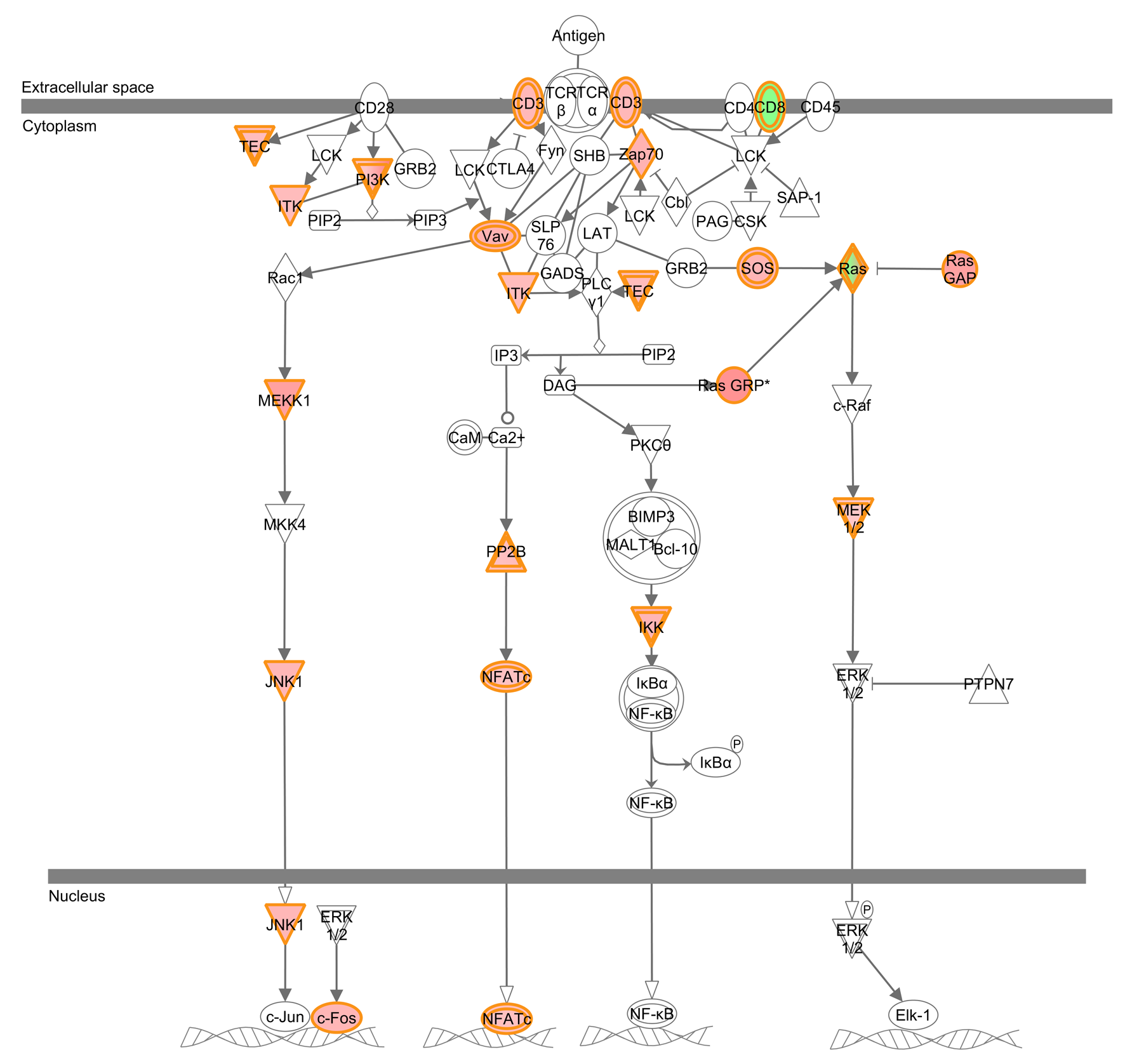

Supplement: Additional file 10 — T cell receptor signalling canonical pathway significantly affected by vaccination. Elements within the IPA canonical pathway T cell receptor signalling differentially expressed after vaccination are indicated. Orange/red elements: upregulated after vaccination, green elements: downregulated after vaccination. Colour intensity reflects the different fold change of expression. [file 1297-9716-44-93-S10.tiff]

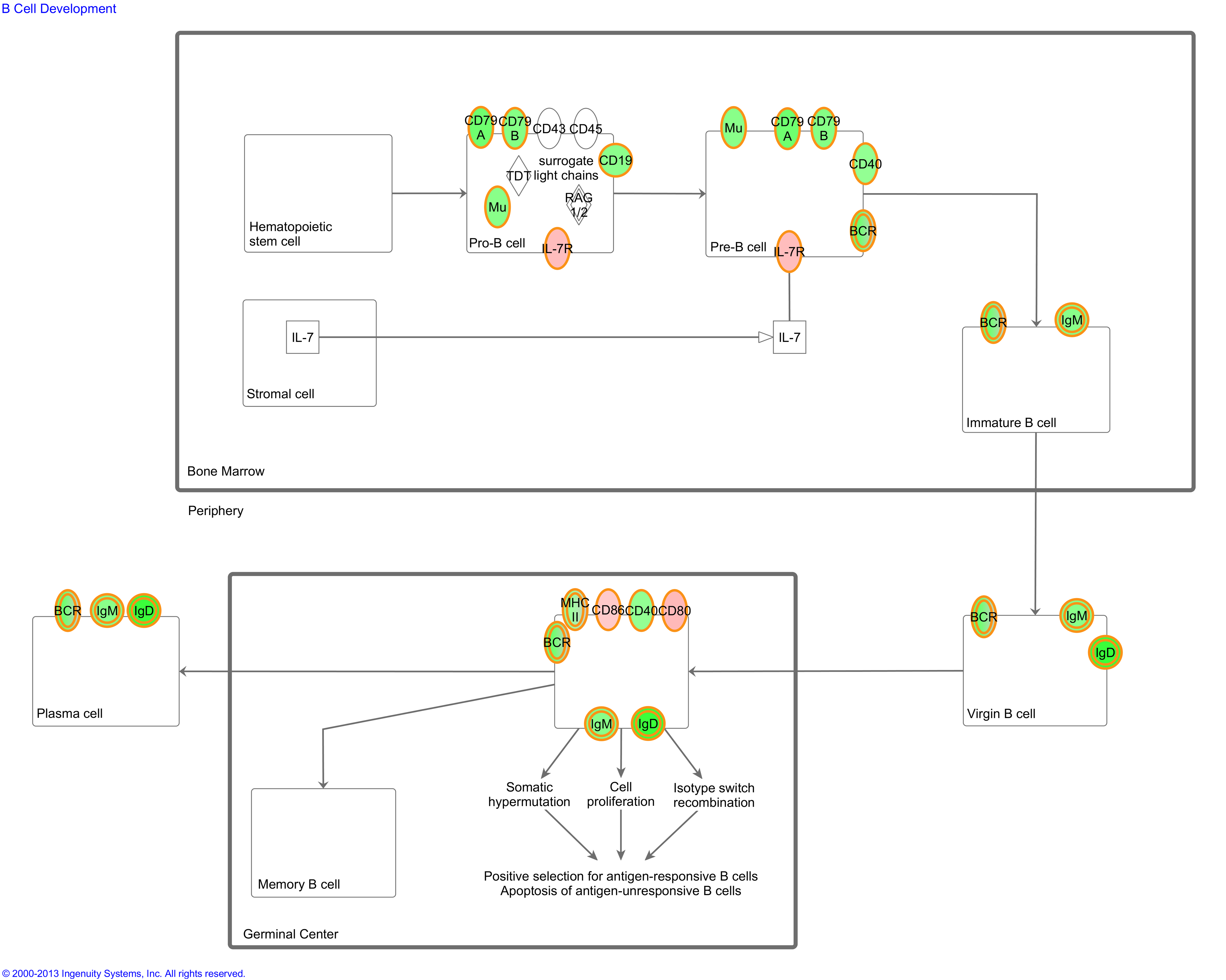

Supplement: Additional file 11 — B cell development canonical pathway significantly affected by vaccination. Elements within the IPA canonical pathway B cell development differentially expressed after vaccination are indicated. Orange/red elements: upregulated after vaccination, green elements: downregulated after vaccination. Colour intensity reflects the different fold change of expression. [file 1297-9716-44-93-S11.tiff]

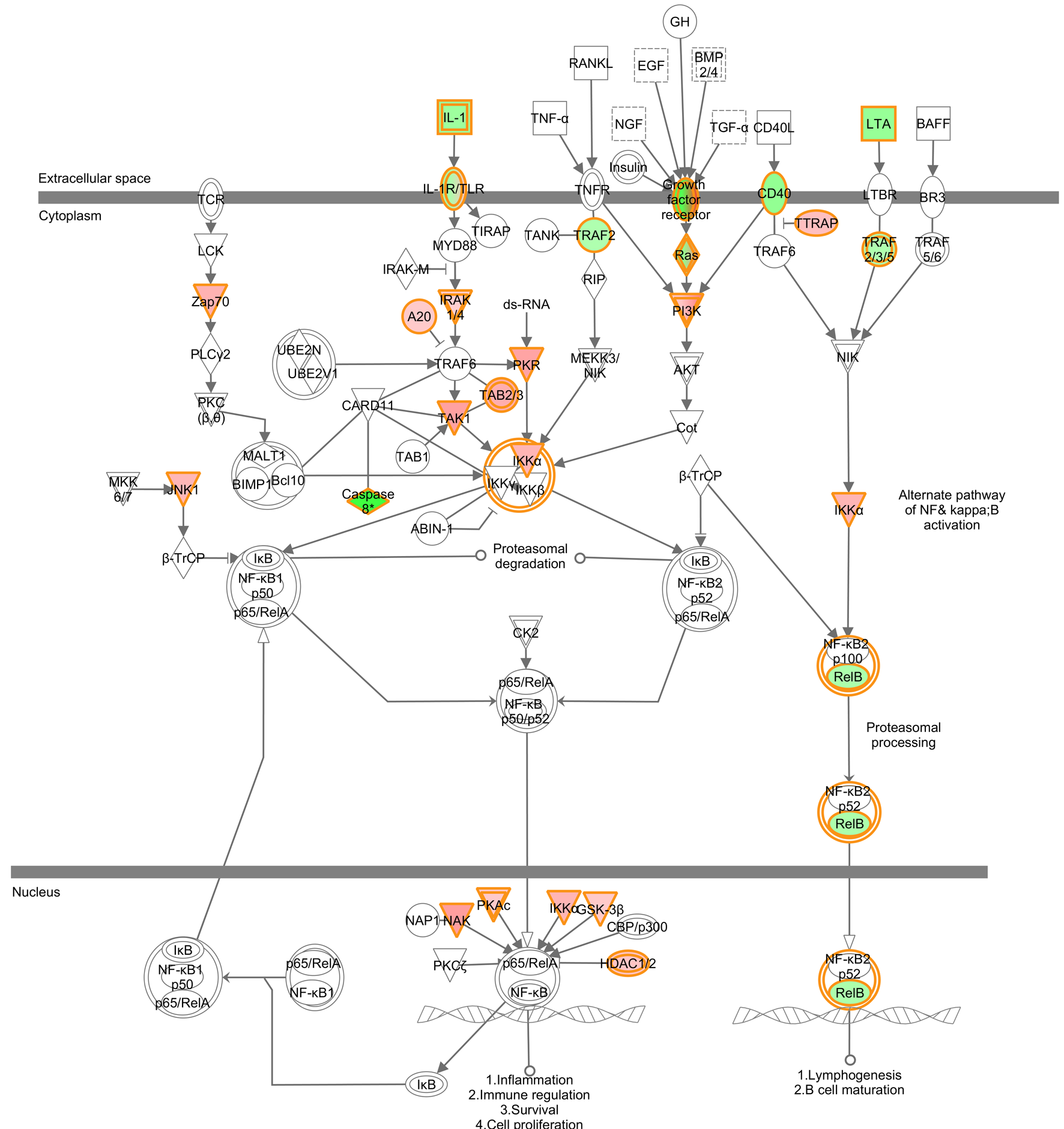

Supplement: Additional file 12 — NFκB signalling canonical pathway significantly affected by vaccination. Elements within the IPA canonical pathway NFκB signalling differentially expressed after vaccination are indicated. Orange/red elements: upregulated after vaccination, green elements: downregulated after vaccination. Colour intensity reflects the different fold change of expression. [file 1297-9716-44-93-S12.tiff]
